# ClC-3 Expression and Its Association with Hyperglycemia Induced HT22 Hippocampal Neuronal Cell Apoptosis

**DOI:** 10.1155/2016/2984380

**Published:** 2016-01-26

**Authors:** Feiyan Fan, Tao Liu, Xin Wang, Dongni Ren, Hui Liu, Pengxing Zhang, Zhen Wang, Nan Liu, Qian Li, Yanyang Tu, Jianfang Fu

**Affiliations:** ^1^Department of Experimental Surgery, Tangdu Hospital, The Fourth Military Medical University, Xi'an 710038, China; ^2^Department of Dermatology, Tangdu Hospital, The Fourth Military Medical University, Xi'an 710038, China; ^3^Department of Neurosurgery, Brigham and Women's Hospital, Harvard Medical School, Boston, MA 02115, USA; ^4^Department of Endocrinology, Xijing Hospital, The Fourth Military Medical University, Xi'an 710032, China

## Abstract

Although apoptosis plays an important role in the development of Diabetic Encephalopathy (DE), the underlying molecular mechanisms remain unclear. With respect to this, the present work aims to study the variation in chloride/proton exchanger ClC-3 expression and its association with HT22 hippocampal neuronal apoptosis under hyperglycemic condition* in vitro*. The cells were stimulated with added 0, 5, or 25 mM glucose or mannitol for up to 72 hours before assessing the rate of ClC-3 expression, cell viability, and apoptosis. In a consecutive experiment, cells received chloride channel blocker in addition to glucose. The rate of cellular death/apoptosis and viability was measured using Flow Cytometry and MTT assay, respectively. Changes in ClC-3 expression were assessed using immunofluorescence staining and western blot analysis. The results revealed a significant increase in cellular apoptosis and reduction in viability, associated with increased ClC-3 expression in high glucose group. Osmolarity had no role to play. Addition of chloride channel blocker completely abolished this effect. Thus we conclude that, with its increased expression, ClC-3 plays a major role in hyperglycemia induced hippocampal neuronal apoptosis. To strengthen our understanding of this aforesaid association, we conducted an extensive literature search which is presented in this paper.

## 1. Introduction

For a long time, central nervous system (CNS), being an insulin independent organ, was thought to be spared from complications of diabetes mellitus (DM). But, in recent decades, various studies have changed this perception by revealing the degenerative response of CNS in chronic DM condition, a condition referred to as Diabetic Encephalopathy (DE), characterized by cognitive dysfunction and failure of learning and memory [1–4]. Its complex pathogenic mechanisms include protein nonenzyme glycosylation, cerebral vessel impairments, abnormal glucocorticoids, transmitters and Tau protein, cell apoptosis, and oxidative stress in the brain. Recent reports strongly suggest that apoptosis plays an important role in the development of DE. Several studies [5–7] have shown that, in diabetic patients, the apoptosis of hippocampal and cortical neurons increases along with an increase in the number of astroglial cells and early degenerative changes that already occur in neurons. Apoptosis of neuronal cells is associated with the opening of mitochondrial permeability transition pores and the upregulated expression of caspase 3 and caspase 8 [8, 9]. The expression levels of apoptosis-inducing factor, cytochrome c, and Bax also bear a close relation with apoptosis. However, the molecules involved in the regulation of DE-related apoptosis and the related molecular mechanism remain unclear.

Chloride (Cl^−^) channels are transmembrane proteins in biological membranes, which form functional pores and allow the diffusion of negatively charged Cl^−^ ions along the electrochemical gradients. These channels can also conduct other anions including I^−^, Br^−^, NO_3_^−^, and aspartate, glutamate. ClC-3 is a member of such Cl^−^ family of anion channels that functions as chloride/proton exchanger [10, 11] that localizes to early and late endosomes as well as to synaptic vesicles (SV). It is widely expressed in most of the mammalian tissues, with a prominent expression in the brain, especially in hippocampal and olfactory bulb neurons [12, 13]. Members of this family have been implicated in having roles in diverse cellular functions such as cellular excitability, synaptic transmission, apoptosis, cell cycle, and blood pressure regulation [14–18]. With respect to its involvement in hippocampal apoptosis a study reports that genetic disruption of ClC-3 chloride/proton exchanger causes postnatal hippocampal neuronal apoptosis/degeneration, making the ClC-3 knockout mouse a unique model in the study of neurodegenerative disease [19]. Another study (*in vitro*) indicates that increased activities of the ClC-3, induced by nitric oxide, may also be involved in hippocampal neuronal apoptosis [20]. In continuation with this, there are no clear studies to predict the role of ClC-3 in neuronal apoptosis under DE condition.

Thus, the present study intends to determine the expression levels of ClC-3 in the hippocampal neurons under hyperglycemic conditions and its associated cellular apoptosis, along with an extensive literature search to better understand the possible molecular mechanisms underlying the results, during development of DE.

## 2. Materials and Methods

### 2.1. HT22 Cells under Hyperglycemic Stimulation

#### 2.1.1. Cell Culture and Treatment

The HT22 hippocampal neuronal cell line, subcloned from parent HT4 cells, was used in this study. This cell line was a generous gift from Department of Anesthesia, Tangdu Hospital, The Fourth Military Medical University, China. Previously HT22 cells have served as a successful* in vitro* model in diabetes associated hippocampal oxidative stress and neurotoxicity studies making them a considerable model for this study [21–23]. The cells were cultured in high glucose DMEM medium (HyClone, Utah, USA) containing 10% fetal bovine serum (FBS) and 1% penicillin-streptomycin (Gibco, California, USA) and maintained under 5% CO_2_ at 37°C. Optimal growth and survival rate of HT22 cells require 25 mM basal glucose. Hence, high glucose DMEM medium was used, as it contains 25 mM glucose and meets these metabolic requirements. After reaching 80% confluency, the cells were trypsinized and replated with a fresh culture medium containing additional 5 mM glucose (total 30 mM glucose; low glucose group) or 25 mM glucose (total 50 mM glucose; high glucose group) (Sigma, Missouri, USA) and cultured for 24, 48, and 72 hours. Control group did not receive any additional glucose (total 25 mM glucose; control group). This aforesaid model has been previously used to mimic hyperglycemic condition [24]. Hence, same model was adopted in our experiment to mimic hyperglycemia.

#### 2.1.2. MTT Assay for Cell Viability

MTT analysis was used to evaluate the impact of glucose on the survival rate of HT22 cells. Cells (seeding density: 4 × 10^3^/well) were seeded in 96-well cell culture plate and cultured with different concentrations of glucose, as explained above. At 24, 48, and 72 h after incubation with glucose, culture medium was replaced with MTT medium, containing 20 *μ*L sterile MTT dye (5 mg/mL), and further incubated at 37°C for 4 h. 100 *μ*L of DMSO (Amresco, Ohio, USA) was mixed into the medium, for the last 10 min of this incubation time, before reading the plate. Microplate reader was used to measure the optical density at 570 nm wavelength. In each group, six samples were measured to get the mean value. The cell viability was calculated by dividing the optical density (OD) value of experimental group with mean OD of normal control group and multiplying by 100.

#### 2.1.3. Flow Cytometric Analysis for Cellular Apoptosis and Death Rate

The cell death and apoptotic rate were measured using Flow Cytometry. At 24 h, after treatment with glucose, HT22 cells were washed with PBS and collected using 0.25% trypsin. After washing with cold PBS, the cells were resuspended in 100 *μ*L of binding buffer containing 5 *μ*L of Annexin V-FITC and 5 *μ*L of PI (propidium iodide). Then cells were gently vortexed and incubated for 30 min at room temperature. Care was taken to avoid light exposure during the procedure. 400 *μ*L of binding buffer was added to each tube. Cells were collected using a flow cytometer and analyzed with Win MDI2.9 software. The obtained results were statistically analyzed.

#### 2.1.4. Western Blot Analysis

Samples from different groups, at 24 h, were lysed in RIPA buffer with 1 : 100 protease inhibitor, on ice for 30 min. Methodically collected cells were centrifuged at 4°C at 13,200 rpm for 15 min; the supernatant was harvested as the total cellular protein extracts and stored at −80°C, until further use. The total protein concentration was determined using BCA assay. Running samples were prepared by adding 5x sample buffer and boiled at 100°C for 5 min to fully denature the proteins. Proteins were separated by SDS-polyacrylamide gel electrophoresis (SDS-PAGE) and transferred to PVDF membrane (Merck, Darmstadt, Germany). Membranes were blocked with 5% nonfat milk in TBST (Tris-Buffered Saline and Tween 20) for 1 h, followed by an overnight incubation with ClC-3 primary antibody (Abcam, Cambridge, UK) at 4°C. Protein bands were detected by incubation with horseradish peroxidase-conjugated goat anti-rabbit IgG (1 : 2000 dilutions) for 1 h and visualized using Electrochemiluminescence (ECL). *β*-actin was used as an internal control. The integrated optical density of positive bands in western blot results was assessed using Image Pro Plus image analysis software and was then statistically analyzed.

#### 2.1.5. Osmotic Control

The role of osmolarity in the observed phenomenon was analyzed by using a biologically inert polysaccharide, mannitol, as an osmotic control. Briefly, trypsinized and replated cells were fed with fresh culture medium containing 5 mM mannitol (total osmolarity from glucose + mannitol = 25 + 5 = 30 mM; mannitol low osmolarity group), 25 mM mannitol (total osmolarity from glucose + mannitol = 25 + 25 = 50 mM; mannitol high osmolarity group), 5 mM glucose (total osmolarity from glucose + glucose = 25 + 5 = 30 mM; glucose low osmolarity group), or 25 mM glucose (total osmolarity from glucose + glucose = 25 + 25 = 50 mM; glucose high osmolarity group). No mannitol or additional glucose was added to control group (total osmolarity from glucose = 25 mM; control). The cells were cultured for 24, 48, and 72 hrs before being subjected to MTT assay to assess the change in cell viability. The change in ClC-3 expression was analyzed in 24 hrs samples by western blot analysis.

#### 2.1.6. Immunocytochemistry

Cells from different groups, terminated at 24 h, were fixed using 4% paraformaldehyde for 15 min at 4°C. After washing with PBS for three times, cells were permeabilized with 0.1% Triton X-100 for 10 min. Following several washes in PBS, cells were blocked with 5% goat serum for 1 h and incubated with anti-ClC-3 primary antibody (ab86192), overnight at 4°C. The following day, cells were washed several times in PBS and FITC-labeled goat anti-rabbit IgG (ZSGB-BIO, Beijing, China) diluted in antibody diluent was added and incubated for 1 h at 37°C. Cell nuclei were stained with DAPI for 10 min. The images were captured under an immunofluorescence microscope. In addition to qualitative analysis, images were also quantified for the difference in the intensity of the antibody uptake, using Image Pro Plus image analysis software, the results of which were statistically analyzed.

#### 2.1.7. (HT22 Cells + Hyperglycemic Stimulation) under the Influence of Chloride Channel Blocker (NPPB)

Further, a follow-up experiment was conducted to assess the relation between the ClC-3 expression rate in HT22 cells and their survival rate under the influence of hyperglycemic conditions. This was accomplished by using a chloride channel blocker 5-nitro-2,3-(phenylpropylamino)-benzoic acid (NPPB) to block the endogenous anion channels upregulated by ClC-3. HT22 cells, cultured as detailed above, received an additional dose of 0.1 mM NPPB in addition to above-mentioned concentrations of glucose. Under these conditions, the cells were cultured for 24 h at the end of which the ClC-3 expression and cellular survival rate were quantified using western blot analysis and MTT assay, respectively. The procedures were kept similar to those explained above.

### 2.2. Statistical Analysis

All the experiments were performed with a sample size of six (*n* = 6) and data are presented as the means ± standard deviation $\left(\overset{-}{x}\pm S\right)$, unless specified. Statistical analysis was performed using the SPSS 11.0 software package. Comparison between two groups was performed using one-way analysis of variance. The qualitative data were compared using the *X* ^2^ test. *P* < 0.05 was considered to be significant.

## 3. Results

### 3.1. HT22 Cells under Hyperglycemic Stimulation

#### 3.1.1. Influence of High Glucose on the Morphology of HT22 Cells

**Figure 1:**
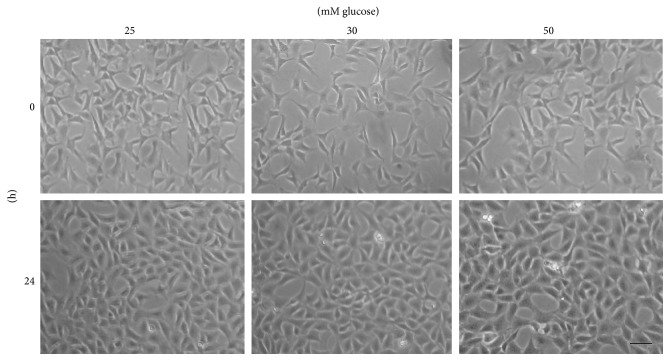
Influence of high glucose on the morphology of HT22 cells as observed after 24 hours of culture: under inverted microscope the 25 mM glucose (control) group and 30 mM glucose stimulation group displayed spindle or multipolar shaped cells with full and transparent cell body with excellent growth, whereas the 50 mM glucose stimulation group showed a reduction in cell attachment and numbers with a significant decrease in light refraction (scale bar 10 *μ*m).

The observation under an inverted light microscope showed a normal morphology of HT22 cells under control groups (no glucose stimulation) and under low glucose group (30 mM glucose stimulation). Specifically, the HT22 cells were spindle or multipolar shaped and possess full and transparent cell body with an excellent growth under both these conditions. On the other hand, the cells under higher glucose (50 mM glucose) stimulation showed a reduction in cell attachment and numbers. Besides, a significant decrease in light refraction was also observed under higher glucose concentration (Figure 1). The optimal cell growth needs basal glucose concentrations of 25 mM, which is found in basal DMEM HG media, as it reflects the higher metabolic rates of neurons. However, supplementing with additional 25 mM glucose to the basal DMEM-HG medium affected the normal growth of HT22 cells, as appreciated under light microscope.

#### 3.1.2. MTT Assay for Cell Viability

**Figure 2:**
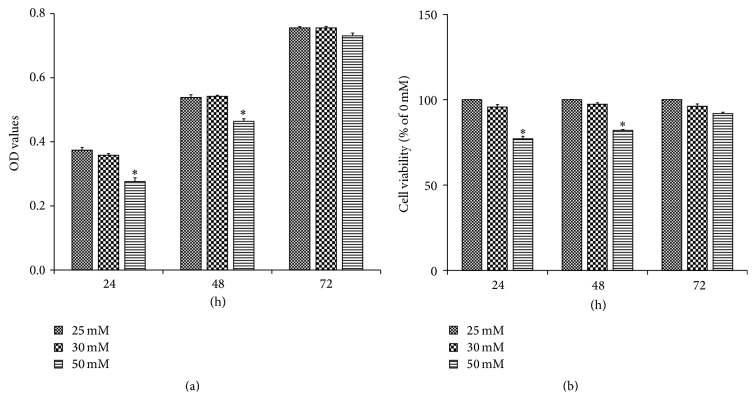
MTT cell viability assay results obtained at 24 h, 48 h, and 72 h displayed in terms of optical density (a) and the percentage of viable cells (% of 0 mM) (b). There was no significant difference between control group and low glucose group (30 mM); however the MTT uptake ability in high glucose group (50 mM) was significantly decreased revealing a significantly lower cell viability (*P* < 0.05).

MTT experiments showed (Figure 2) that the cells were all viable under control group and the low glucose group, with no significant difference between the groups (*P* > 0.05). However, decreased MTT uptake ability by HT22 cells, in high glucose group, revealed a decrease in their viability and survival rate. As compared to the control and low glucose groups, this difference in viability under high glucose group reached statistical significance at 24 and 48 h (*P* < 0.05). Although less viable, the difference at 72 h failed to reach the significance (*P* > 0.05). Since the difference at 24 h was apparently prominent, further experiments were limited to this time point.

#### 3.1.3. Flow Cytometric Analysis for Cellular Apoptosis and Death Rate

**Figure 3:**
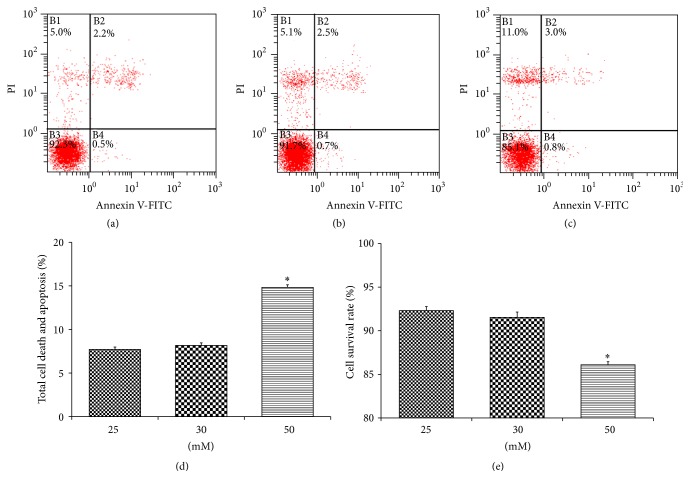
Flow cytometric analysis of apoptotic and dead cells after 24 h of glucose stimulation in control group (a), 30 mM glucose stimulation group (b), and 50 mM glucose stimulation group (c) represented as apoptotic and dead cells (d) and viable cells (e). Results showed a significantly higher apoptotic activity and lower survival rate in high glucose group (*P* < 0.05).

Changes in cell apoptosis and death were observed using FCM (Flow Cytometry) after 24 h of glucose stimulation. The results showed a significantly higher death/apoptotic activity and lower survival rate (*P* < 0.05) of HT22 cells, in high glucose group as compared to the other two groups, after 24 h of glucose stimulation, thus elucidating the early damaging effects of hyperglycemia on the cells and the level of cellular sensitivity to high glucose (Figure 3).

#### 3.1.4. Western Blot Analysis

**Figure 4:**
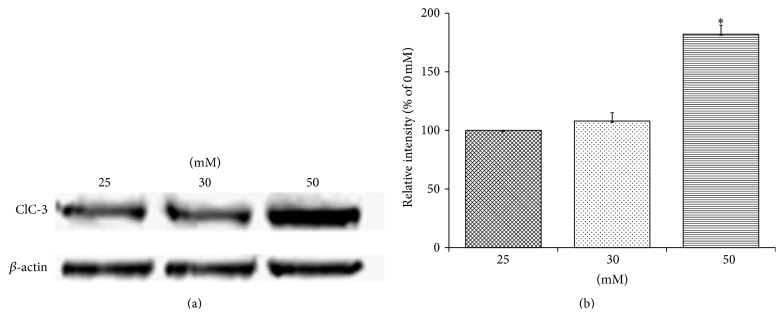
Western blot analysis of HT22 cell lysates from control and low and high glucose groups, at 24 h, for ClC-3 protein expression (a) and quantification of its intensity levels (ratio of ClC-3 band to standard *β*-actin band) (b) showed a significant increase in the expression level of ClC-3 in high glucose group (*P* < 0.05).

Total protein concentration, of cells after 24 h of culture, was determined using BCA assay and proteins were subjected to western blot analysis. As shown in Figure 4, ClC-3 protein expression levels (intensity ratio of ClC-3 band to the standard band *β*-actin) were analyzed and the results were expressed as percentage of control ± SEM (*n* = 5). The results showed a significant increase (*P* < 0.05) in the expression level of ClC-3 in high glucose group as compared to the other two groups.

#### 3.1.5. Osmotic Control

**Figure 5:**
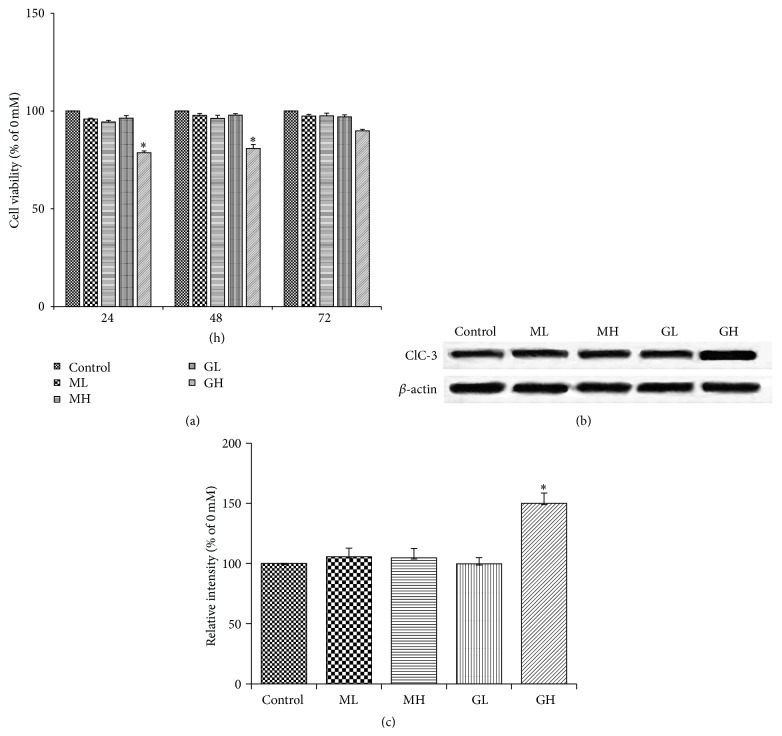
Osmotic control experiment: MTT cell viability assay, results of glucose and mannitol (osmotic control) groups obtained at 24 h, 48 h, and 72 h displayed in terms of the percentage of viable cells (% of 0 mM) (a). Western blot analysis of HT22 cell lysates from control, mannitol, and glucose groups, at 24 h, for ClC-3 protein expression (b) and quantification of their respective intensity levels (ratio of ClC-3 band to standard *β*-actin band) (c) showed a significant increase in the expression level of ClC-3 in high glucose group (*P* < 0.05). Control: normal osmolarity (25 mM) group, ML: mannitol low osmolarity (30 mM) group, MH: mannitol high osmolarity (50 mM) group, GL: glucose low osmolarity (30 mM) group, and GH: glucose high osmolarity (50 mM) group.

The effect of change in osmolarity of the medium on the above results was assessed by using mannitol as an osmotic control. MTT assay revealed no noticeable change in the HT22 cell survival in response to change in the osmolarity while reproducing a significant decrease in their viability under high glucose group (Figure 5(a)). Also, increase in osmolarity failed to elicit changes in the ClC-3 expression in HT22 cells while the same was found significantly increased in high glucose group (Figures 5(b) and 5(c)). These results rule out the effect of osmolarity indicating glucose, at the assessed concentration, as the cause of the observed phenomenon.

#### 3.1.6. Immunocytochemistry: The Localization and Expression Levels of ClC-3 in HT22 Cells

**Figure 6:**
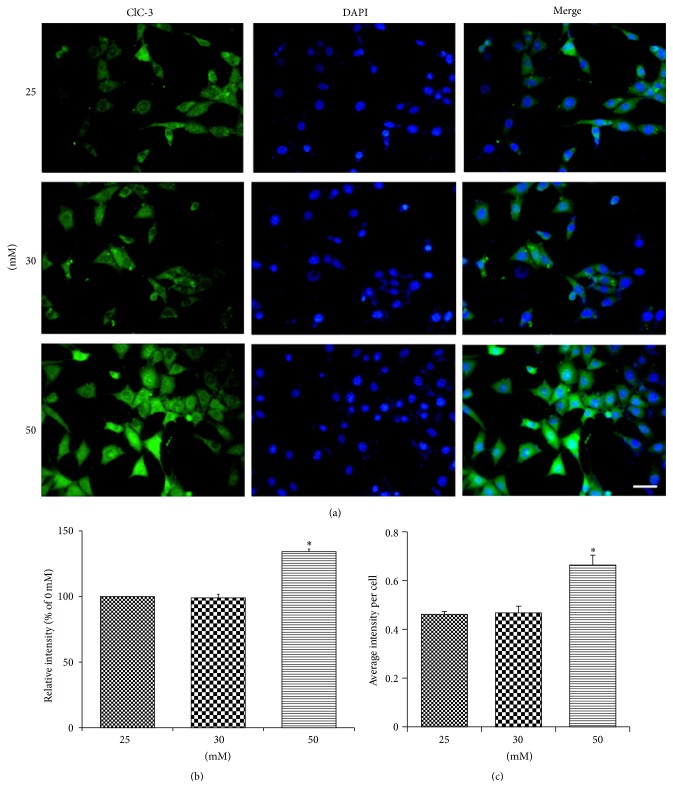
Immunofluorescence staining of HT22 cells to determine the ClC-3 protein expression, stimulated under different glucose concentrations (a), intensity of images, as measured using Image Pro Plus 6.0 (b), and the “average intensity per cell” calculated by dividing the intensity of the image by its total number of cells (c). HT22 cells expressed ClC-3 protein marker. The intensity of the images and the intensity per cell in the high glucose (50 mM) group were significantly higher than those of control and low glucose (30 mM) group (*P* < 0.05).

ClC-3 was mainly found distributed in the cytoplasm which was evident from immunocytochemistry (Figure 6(a)). Relative fluorescence intensity (% of 0 mM) per image, measured using Image Pro Plus 6.0, was found to be significantly higher in the high glucose group compared to the other two groups (Figure 6(b)). The positive ClC-3 fluorescence intensity measured per image was divided by the total number of cells in the image to obtain the average intensity per cell, which was significantly higher (*P* < 0.05) in the high glucose group than the other two groups (Figure 6(c)). This shows that, in each cell, the level of ClC-3 expression gets significantly increased, compared to its normal levels, in response to high glucose stimulation.

#### 3.1.7. (HT22 Cells + Hyperglycemic Stimulation) under the Protection of Chloride Channel Blocker (NPPB)

**Figure 7:**
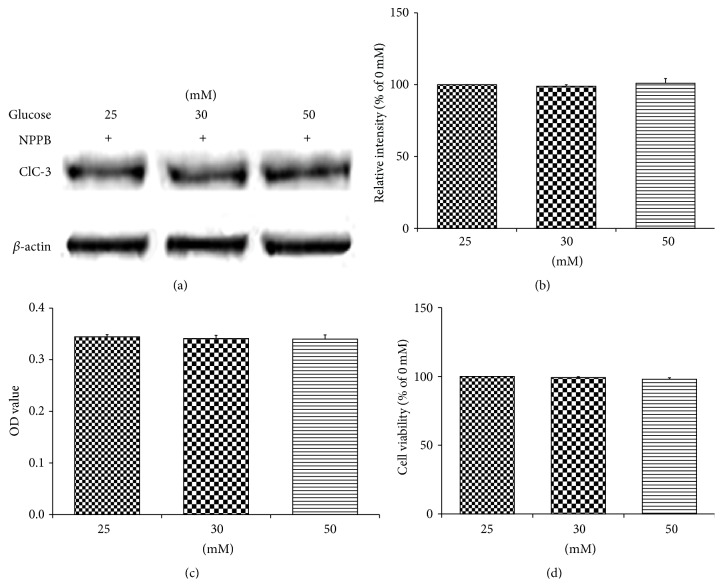
Western blot analysis of HT22 cell lysates from control and low and high glucose groups, in the presence of 0.1 M NPPB, at 24 h, stained for ClC-3 protein expression (a) reveals no change in the total ClC-3 expression, in terms of their intensity, among groups (b). MTT assay of HT22 cells, cultured under same conditions, showed no significant changes in the cell viability among the groups (c and d).

NPPB was used to block the ClC-3 upregulated, sensitive endogenous anion channels. Surprisingly, it was found that the total ClC-3 expression of HT22 cells under 50 mM glucose stimulation was also downregulated to its normal levels, as evidenced by western blot analysis. No changes in the total ClC-3 expression of control and 30 mM glucose groups were observed (Figures 7(a) and 7(b)). Including the high glucose group, the expression level of ClC-3 remained constant across all the groups. Interestingly MTT assay showed no significant changes in the cell viability between the groups, including high glucose group (Figures 7(c) and 7(d)). In fact, the cell viability rate was very similar across the groups, indicating no cell deaths under high glucose stimulation. Considering the low viability rate of HT22 cells under high glucose in previous experiment, here, it was exciting to see how the blocking of ClC-3 upregulated NPPB sensitive endogenous anion channels along with a downregulation of ClC-3 itself to normal levels successfully and completely abolished the hyperglycemia induced hippocampal neuronal apoptosis. This result established a strong relation between increased ClC-3 expression and HT22 cell survival rate, under hyperglycemic condition. Also it reveals the significant role of ClC-3 protein in mediating the hyperglycemia induced hippocampal neuronal apoptosis pathway.

## 4. Discussion

In the light of our finding, we did a thorough literature search to understand the possible underlying pathway, involved in hyperglycemia induced ClC-3 mediated hippocampal neuronal apoptosis, which is presented below.

In the intervening years, accumulating evidence shows that the ClC-3 family of chloride/proton exchanger plays a fundamental role in normal excitatory and inhibitory neurotransmission in central nervous system via various pathways. Various theories accounting for ClC-3's role in neuroregulatory functions are proposed to explain why ClC-3 triggers seizures and neurodegeneration. It was suggested to mediate swelling-activated plasma membrane currents via Cl^−^ currents or Ca_2_-activated Cl^−^ currents in plasma membranes [19, 25]. ClC-3^−/−^ mice did not lack both of these currents, suggesting that loss of ClC-3 does not affect these currents [19, 26, 27]. However, its other key function is acidification of synaptic vesicles, which is reported to be affected by ClC-3. When acidification of synaptic vesicles is impaired, there is severe postnatal degeneration of the retina and the hippocampus [19]. Changes in ClC-3 expression levels also have been associated with hippocampal neuronal degeneration.

A study suggests that ClC-3 in PC12 cells mediates Thapsigargin- (TG-) induced apoptosis. TG decreased the cell proliferation and increased the cell apoptotic population with the decrease in endogenous ClC-3 protein expression. On the other hand, the ClC-3 cDNA transfection reversed these effects induced by TG. Thus, it appears that ClC-3 mediates both cell proliferation and apoptosis through accelerative and inhibitory fashions, respectively [28]. In another study, genetic disruption of ClC-3 in mice results in severe neurodegeneration in central nervous system via excessive release of glutamate [29]. There is a hypothesis that ClC-3 plays a key role in neuronal cell function by regulating membrane excitability by protein kinase C and may have a role in short term memory loss. Stobrawa et al. [19] showed that the ClC-3 knockout mice congenitally displayed a near absence of the hippocampus and of photoreceptors, defects that might be associated with loss of long-term memory function.

ClC-3 is necessary for the activation of smooth muscle cells as well [30]. Deficiency of ClC-3 markedly reduces neointimal hyperplasia following vascular injury. A study carried out by Takahashi et al. [31] states that the Cl^−^ channel dependent apoptosis of ischemic myocardial cells, in transgenic mouse, was abolished with the use of chloride channel blockers like DIDS and NPPB. These findings identify ClC-3 and other Cl^−^ channels as a novel target for the prevention of cellular apoptosis in inflammatory and proliferative vascular diseases [30].

The study on ClC-3 expression is also reported in diabetic condition. A group identified that ClC-3 is related to preadipocyte apoptosis induced by palmitate, both* in vitro* and* in vivo* (type 2 diabetic mice). ClC-3 knockout significantly attenuated preadipocyte apoptosis and the above metabolic disorders in type II diabetic mice. These data demonstrated that ClC-3 deficiency protects preadipocytes against palmitate-induced apoptosis via suppressing ER stress and also suggested that ClC-3 may play a role in regulating cellular apoptosis and disorders of glucose and lipid metabolism during type II diabetes mellitus [32]. ClC-3 is also proposed to have roles in neutrophil and smooth muscle reactive oxygen species generation [33, 34], and in cell insulin secretion [34]. This gives us a clue that the oxidative stress and ROS generation would be a major pathway in ClC-3 mediated effect on diabetes and its related neurodegenerative issues. Deriy et al. [35] identified that ClC-3 knockout mice contained higher amounts of proinsulin than in granules from normal mice. They found a slowdown in conversion of proinsulin to insulin inside the granules in the ClC-3 knockout mice. Though the lack of ClC-3 is reported to have an effect on diabetes and its neurocognitive diseases, literature does report on the effect of increased expression of ClC-3 under diabetic condition in causing other problems like cataract. The upregulation of ClC-3 and Na(+)/Ca(2+) exchanger proteins during the early stages of diabetes and its prevention by antioxidants suggest that the protein regulating ion transport may have a pathophysiological role in the development of diabetic cataracts. Besides, a recent study [36] indicates that the increased activities of ClC-3 may be involved in hippocampal neuronal apoptosis induced by nitric oxide. According to their result, after hippocampal neuronal injury, ClC-3 expression becomes upregulated. Simultaneously, Cl^−^ and K^+^ efflux lead to massive outflow of intracellular water, which reduces the cell volume and then triggers neuronal apoptosis [20].

Although the role of ClC-3 has been reported to study the diabetes related complications and its effect on hippocampal neurons, the correlation of ClC-3 and its mediated hippocampal loss under hyperglycemic conditions is not yet reported. Studies report that hyperglycemia leads to oxidative stress and apoptosis of neurons. It is demonstrated that the complication is mainly caused by high glucose that leads to nonenzymatic glycation, redox stress, aldose reductase activation, and diacylglycerol-protein kinase C (DAG-PKC) pathway activation [37]. As ClC-3 is associated with the PKC mediated pathways, ROS activation via nitric oxide, thereby leading to increased cellular oxidative stress, we could hypothesize that ClC-3 regulation could play a key role in affecting hippocampal neurons, under hyperglycemic conditions, via these pathways. Inhibition of ROS, as well as maintenance of euglycemia, may restore metabolic and vascular imbalances and block both the initiation and progression of neuropathy [37–39]. However, the underlying mechanism is not much clear.

Oxidative stress has been widely considered as a key player in the adverse effects of hyperglycemia to various tissues, including neuronal cells. Oxidative stress diminishes the activity of endogenous antioxidant enzyme defense system (SOD, catalase, and glutathione peroxidase), playing a significant protective role. It has been demonstrated that, under hyperglycemic conditions, free radicals are produced in dorsal root ganglion neurons leading to mitochondrial dysfunction [40, 41]. They have also demonstrated that mitochondrial dysfunction and apoptosis seen in diabetic rats can be recapitulated in an* in vitro* cell culture model [24, 42, 43].

In our experiment, we speculated that ClC-3 could have an association with hippocampal neuronal apoptosis under hyperglycemic conditions, which is a first of its kind work. Hence, we used HT22 cells, a widely studied* in vitro* model to study the status of hippocampal neurons in diabetes mellitus, by determining its effect and correlation with high glucose conditions. HT22 cells were exposed to different concentrations of glucose for 24 h, 48 h, and 72 h. This experiment showed a reduction in survival rates of neuronal cells both at 24 h and at 48 h. The effect of 24 h stimulation had a slightly greater effect on cell survival rates, suggesting that stimulation of neuronal cells by high glucose can induce apoptotic changes at a very early stage. Neuronal apoptosis is a genetically regulated autonomic programmed cell death process. Our study also used FCM with FITC-Annexin V and PI staining as a detection method to observe the effects on the apoptosis and death of HT22 hippocampal neuronal cells under high glucose stimulation for 24 h. The results showed that cell apoptosis and death rates both increased after high glucose stimulation as compared to control and low glucose stimulation. These results, agreeing with other studies, suggest that the high glucose can induce apoptosis and death of hippocampal neuronal cells and reduce cell growth and survival rates.

Our immunofluorescence staining results revealed that ClC-3 was expressed by HT22 cells. Significant localization was observed in the cell cytoplasm; in addition, the expression levels of ClC-3 significantly increased after stimulation with high glucose. The western blot results using total protein extracts from cells stimulated with high glucose for 24 h showed that the ClC-3 expression level had increased compared to the control and low glucose groups. This result suggested that high glucose could induce an increase in ClC-3 expression in hippocampal neuronal cells, correlating with its already established ability to induce neuronal apoptosis and death. Any possible role of osmolarity, both in decreased cell viability and in increased ClC-3 expression, was ruled out through osmotic control experiments using mannitol. On the other hand, when the ClC-3 upregulated NPPB sensitive endogenous anion channels were blocked using a chloride channel blocker, NPPB, hyperglycemia induced hippocampal neuronal apoptosis/death was abolished, thus revealing the significance of ClC-3 protein and its crucial role during the process. We also found that addition of NPPB downregulated the increased ClC-3 levels back to its normal values. This was surprising, especially under the established knowledge that ClC-3 is not sensitive to NPPB. A thorough literature search in this regard reveals a fact that the existence of two variants of ClC-3 has been identified, long form and short form [44]. Of these, unlike short form the long form ClC-3 is found to be sensitive to NPPB [45]. Considering this phenomenon we may hypothesize that the upregulated ClC-3, seen in hippocampal cells (HT22 cells) in response to high glucose stimulation, is probably the long form variant which might be responsible for decrease in cell viability and may have responded to NPPB. And the fact that the normal level of ClC-3 expression was maintained in all the groups may indicate that, under normal condition, short form ClC-3 may predominate in these cells and thus was unaffected by NPPB. This new and interesting hypothesis definitely needs further evaluation. Our literature search also substantiates the ability of increased expression of ClC-3 in inducing cellular apoptosis and suggests nitric oxide pathway via ROS activation and PKC mediated pathway as the possible pathways involved in ClC-3 mediated neurodegenerative issues in diabetes, which needs to be verified further. Thus, our study substantiates our hypothesis that increased expression of ClC-3 plays a crucial role in mediating the hyperglycemia induced hippocampal neuronal apoptosis, thus assisting in the development of Diabetic Encephalopathy and other associated neurodegenerative complications.

A study reports that differentiated HT22 cells possess postmitotic neuronal properties and thus represent a better* in vitro* model for hippocampal neuronal studies than undifferentiated cells. The study noted a significant susceptibility of differentiated HT22 cells to glutamate induced excitotoxicity compared to its undifferentiated variant. Also only differentiated but not undifferentiated HT22 cells showed a positive response to the protective effects of N-methyl-D-aspartate receptor antagonists against glutamate, marking the difference amongst the two [46]. In the present study we have used the HT22 cells in their undifferentiated form. Differentiated HT22 cells may establish a better synapse and thus show a better ClC-3 expression/activity which may vary its response to high glucose. Hence differentiated HT22 cells may serve as a better model for future studies, which is recommended.

## 5. Conclusion

In DM, high glucose can induce changes in blood osmolarity and in Cl^−^ channel activity and as our study found, it could increase ClC-3 expression in HT22 hippocampal neuronal cells, which had inhibitory effects on their growth and survival. As evidenced by our literature search, the possible mechanisms by which ClC-3 acts on hippocampal neurons may be associated with ROS and PKC mediated oxidative stress pathways. Thus, our research work stands as a pilot study, a first of its kind finding correlation between hyperglycemia induced increased ClC-3 expression in hippocampal neurons and their apoptosis. However it warrants further studies to establish these results at organismal level and also to identify the exact underlying molecular mechanisms. Also the possible change in the response of long and short form variant of ClC-3 to glucose warrants further investigation. We hope the results from our study act as the basis for such future studies, the results of which may provide us with novel target molecules and pathways, aiding in slowing/preventing development of Diabetic Encephalopathy and other neurocognitive issues related to diabetes.

## References

[1] Mijnhout G. S., Scheltens P., Diamant M. (2006). Diabetic encephalopathy: a concept in need of a definition. *Diabetologia*.

[2] Ola M. S., Aleisa A. M., Al-Rejaie S. S. (2014). Flavonoid, morin inhibits oxidative stress, inflammation and enhances neurotrophic support in the brain of streptozotocin-induced diabetic rats. *Neurological Sciences*.

[3] Liu J., Wang S., Feng L. (2013). Hypoglycemic and antioxidant activities of paeonol and its beneficial effect on diabetic encephalopathy in streptozotocin-induced diabetic rats. *Journal of Medicinal Food*.

[4] Meng X., Wang X., Tian X., Yang Z., Li M., Zhang C. (2014). Protection of neurons from high glucose-induced injury by deletion of MAD2B. *Journal of Cellular and Molecular Medicine*.

[5] den Heijer T., Vermeer S. E., van Dijk E. J. (2003). Type 2 diabetes and atrophy of medial temporal lobe structures on brain MRI. *Diabetologia*.

[6] Alvarez E. O., Beauquis J., Revsin Y. (2009). Cognitive dysfunction and hippocampal changes in experimental type 1 diabetes. *Behavioural Brain Research*.

[7] Saravia F. E., Beauquis J., Revsin Y., Homo-Delarche F., de Kloet E. R., De Nicola A. F. (2006). Hippocampal neuropathology of diabetes mellitus is relieved by estrogen treatment. *Cellular and Molecular Neurobiology*.

[8] Jentsch T. J., Stein V., Weinreich F., Zdebik A. A. (2002). Molecular structure and physiological function of chloride channels. *Physiological Reviews*.

[9] Velier J. J., Ellison J. A., Kikly K. K., Spera P. A., Barone F. C., Feuerstein G. Z. (1999). Caspase-8 and caspase-3 are expressed by different populations of cortical neurons undergoing delayed cell death after focal stroke in the rat. *Journal of Neuroscience*.

[10] Guzman R. E., Grieschat M., Fahlke C., Alekov A. K. (2013). ClC-3 is an intracellular chloride/proton exchanger with large voltage-dependent nonlinear capacitance. *ACS Chemical Neuroscience*.

[11] Guzman R. E., Miranda-Laferte E., Franzen A., Fahlke C. (2015). Neuronal ClC-3 splice variants differ in subcellular localizations, but mediate identical transport functions. *The Journal of Biological Chemistry*.

[12] Schmieder S., Lindenthal S., Ehrenfeld J. (2001). Tissue-specific N-glycosylation of the ClC-3 chloride channel. *Biochemical and Biophysical Research Communications*.

[13] Kawasaki M., Uchida S., Monkawa T. (1994). Cloning and expression of a protein kinase C-regulated chloride channel abundantly expressed in rat brain neuronal cells. *Neuron*.

[14] Farmer L. M., Le B. N., Nelson D. J. (2013). CLC-3 chloride channels moderate long-term potentiation at Schaffer collateral-CA1 synapses. *Journal of Physiology*.

[15] (2009). Chloride channels. *British Journal of Pharmacology*.

[16] Duan D. D. (2010). Volume matters: novel roles of the volume-regulated ClC-3 channels in hypertension-induced cerebrovascular remodeling. *Hypertension*.

[17] Nilius B., Droogmans G. (2003). Amazing chloride channels: an overview. *Acta Physiologica Scandinavica*.

[18] Wang X. Q., Deriy L. V., Foss S. (2006). CLC-3 channels modulate excitatory synaptic transmission in hippocampal neurons. *Neuron*.

[19] Stobrawa S. M., Breiderhoff T., Takamori S. (2001). Disruption of ClC-3, a chloride channel expressed on synaptic vesicles, leads to a loss of the hippocampus. *Neuron*.

[20] Xu L., Zhang S., Fan H. (2013). ClC-3 chloride channel in hippocampal neuronal apoptosis. *Neural Regeneration Research*.

[21] Račková L., Šnirc V., Jung T., Štefek M., Karasu C., Grune T. (2009). Metabolism-induced oxidative stress is a mediator of glucose toxicity in HT22 neuronal cells. *Free Radical Research*.

[22] Yang Y., Ma D., Xu W. (2016). Exendin-4 reduces tau hyperphosphorylation in type 2 diabetic rats via increasing brain insulin level. *Molecular and Cellular Neuroscience*.

[23] Maher P., Dargusch R., Ehren J. L., Okada S., Sharma K., Schubert D. (2011). Fisetin lowers methylglyoxal dependent protein glycation and limits the complications of diabetes. *PLoS ONE*.

[24] Gaspar J. M., Castilho Á., Baptista F. I., Liberal J., Ambrósio A. F. (2010). Long-term exposure to high glucose induces changes in the content and distribution of some exocytotic proteins in cultured hippocampal neurons. *Neuroscience*.

[25] Huang L., Cao J., Wang H., Vo L. A., Brand J. G. (2005). Identification and functional characterization of a voltage-gated chloride channel and its novel splice variant in taste bud cells. *The Journal of Biological Chemistry*.

[26] Arreola J., Begenisich T., Nehrke K. (2002). Secretion and cell volume regulation by salivary acinar cells from mice lacking expression of the *Clcn3* Cl^−^ channel gene. *The Journal of Physiology*.

[27] Gong W., Xu H., Shimizu T. (2004). ClC-3-independent, PKC-dependent activity of volume-sensitive Cl^−^ channel in mouse ventricular cardiomyocytes. *Cellular Physiology and Biochemistry*.

[28] Zhang H.-N., Zhou J.-G., Qiu Q.-Y., Ren J.-L., Guan Y.-Y. (2006). ClC-3 chloride channel prevents apoptosis induced by thapsigargin in PC12 cells. *Apoptosis*.

[29] Guzman R. E., Alekov A. K., Filippov M., Hegermann J., Fahlke C. (2014). Involvement of ClC-3 chloride/proton exchangers in controlling glutamatergic synaptic strength in cultured hippocampal neurons. *Frontiers in Cellular Neuroscience*.

[30] Chu X., Filali M., Stanic B. (2011). A Critical role for chloride channel-3 (CIC-3) in smooth muscle cell activation and neointima formation. *Arteriosclerosis, Thrombosis, and Vascular Biology*.

[31] Takahashi N., Wang X., Tanabe S. (2005). ClC-3-independent sensitivity of apoptosis to Cl^−^ channel blockers in mouse cardiomyocytes. *Cellular Physiology and Biochemistry*.

[32] Huang Y.-Y., Huang X.-Q., Zhao L.-Y. (2014). ClC-3 deficiency protects preadipocytes against apoptosis induced by palmitate in vitro and in type 2 diabetes mice. *Apoptosis*.

[33] Moreland J. G., Davis A. P., Bailey G., Nauseef W. M., Lamb F. S. (2006). Anion channels, including ClC-3, are required for normal neutrophil oxidative function, phagocytosis, and transendothelial migration. *The Journal of Biological Chemistry*.

[34] Miller F. J., Filali M., Huss G. J. (2007). Cytokine activation of nuclear factor *κ*B in vascular smooth muscle cells requires signaling endosomes containing Nox1 and ClC-3. *Circulation Research*.

[35] Deriy L. V., Gomez E. A., Jacobson D. A. (2009). The granular chloride channel ClC-3 is permissive for insulin secretion. *Cell Metabolism*.

[36] Zhong Z. C., Fan H., Yin J. B., Zheng Y., Xu L., Chang Q. (2011). Changes of chloride channel currents during cultured rat hippocampal neuronal apoptosis and anti-effects of SITS. *Zhengzhou-Daxue-Xuebao/Yixue-Ban*.

[37] Brownlee M. (2001). Biochemistry and molecular cell biology of diabetic complications. *Nature*.

[38] Feldman E. L., Stevens M. J., Russell J. W., Greene D. A., Porte D., Sherwin R. S., Baron A. (2002). Somatosensory neuropathy. *Ellenberg and Rifkin's Diabetes Mellitus*.

[39] Vincent A. M., McLean L. L., Backus C., Feldman E. L. (2005). Short-term hyperglycemia produces oxidative damage and apoptosis in neurons. *The FASEB Journal*.

[40] Schmeichel A. M., Schmelzer J. D., Low P. A. (2003). Oxidative injury and apoptosis of dorsal root ganglion neurons in chronic experimental diabetic neuropathy. *Diabetes*.

[41] Cheng C., Zochodne D. W. (2003). Sensory neurons with activated caspase-3 survive long-term experimental diabetes. *Diabetes*.

[42] Russell J. W., Sullivan K. A., Windebank A. J., Herrmann D. N., Feldman E. L. (1999). Neurons undergo apoptosis in animal and cell culture models of diabetes. *Neurobiology of Disease*.

[43] Russell J. W., Golovoy D., Vincent A. M. (2002). High glucose-induced oxidative stress and mitochondrial dysfunction in nuerons. *The FASEB Journal*.

[44] Shimada K., Li X., Xu G., Nowak D. E., Showalter L. A., Weinman S. A. (2000). Expression and canalicular localization of two isoforms of the ClC-3 chloride channel from rat hepatocytes. *The American Journal of Physiology—Gastrointestinal and Liver Physiology*.

[45] Li X., Shimada K., Showalter L. A., Weinman S. A. (2000). Biophysical properties of ClC-3 differentiate it from swelling-activated chloride channels in Chinese hamster ovary-K1 cells. *The Journal of Biological Chemistry*.

[46] He M., Liu J., Cheng S., Xing Y., Suo W. Z. (2013). Differentiation renders susceptibility to excitotoxicity in HT22 neurons. *Neural Regeneration Research*.

